# Succinate causes pathological cardiomyocyte hypertrophy through GPR91 activation

**DOI:** 10.1186/s12964-014-0078-2

**Published:** 2014-12-24

**Authors:** Carla J Aguiar, João A Rocha-Franco, Pedro A Sousa, Anderson K Santos, Marina Ladeira, Cibele Rocha-Resende, Luiz O Ladeira, Rodrigo R Resende, Fernando A Botoni, Marcos Barrouin Melo, Cristiano X Lima, José M Carballido, Thiago M Cunha, Gustavo B Menezes, Silvia Guatimosim, M Fatima Leite

**Affiliations:** Department of Physiology and Biophysics, Federal University of Minas Gerais, Av. Antônio Carlos 6627, Belo Horizonte, MG - CEP: 31270-901 Brazil; Department of Biochemistry and Immunology, Federal University of Minas Gerais, Av. Antônio Carlos 6627, Belo Horizonte, MG - CEP: 31270-901 Brazil; Department of Physics, Federal University of Minas Gerais, Av. Antônio Carlos 6627, Belo Horizonte, MG - CEP: 31270-901 Brazil; Department of Medicine, Federal University of Minas Gerais, Av. Antônio Carlos 6627, Belo Horizonte, MG - CEP: 31270-901 Brazil; Novartis Institutes for Biomedical Research, Basel, CH-4002 Switzerland; Department of Pharmacology, Ribeirão Preto, Medical School, University of São Paulo, São Paulo, Brazil; Department of Morphology, Federal University of Minas Gerais, Av. Antônio Carlos 6627, Belo Horizonte, MG - CEP: 31270-901 Brazil

**Keywords:** Succinate, Cardiomyocyte, Hypertrophy

## Abstract

**Background:**

Succinate is an intermediate of the citric acid cycle as well as an extracellular circulating molecule, whose receptor, G protein-coupled receptor-91 (GPR91), was recently identified and characterized in several tissues, including heart. Because some pathological conditions such as ischemia increase succinate blood levels, we investigated the role of this metabolite during a heart ischemic event, using human and rodent models.

**Results:**

We found that succinate causes cardiac hypertrophy in a GPR91 dependent manner. GPR91 activation triggers the phosphorylation of extracellular signal-regulated kinase 1/2 (ERK1/2), the expression of calcium/calmodulin dependent protein kinase IIδ (CaMKIIδ) and the translocation of histone deacetylase 5 (HDAC5) into the cytoplasm, which are hypertrophic-signaling events. Furthermore, we found that serum levels of succinate are increased in patients with cardiac hypertrophy associated with acute and chronic ischemic diseases.

**Conclusions:**

These results show for the first time that succinate plays an important role in cardiomyocyte hypertrophy through GPR91 activation, and extend our understanding of how ischemia can induce hypertrophic cardiomyopathy.

**Electronic supplementary material:**

The online version of this article (doi:10.1186/s12964-014-0078-2) contains supplementary material, which is available to authorized users.

## Background

Cardiac hypertrophy is an adaptive response to biomechanical overload or extracellular stimuli and it is associated with augmented risk of heart failure and sudden death [1-3]. At the molecular level, cardiomyocyte hypertrophy is characterized by reinduction of the so-called fetal gene program, leading to upregulation of genes encoding atrial and brain natriuretic peptides, β-myosin heavy chain and skeletal α-actin [4]. At the cellular level, increased cell-size and enhanced protein synthesis are the prominent characteristics [5]. Several pathologies including hypertension and ischemic diseases are known to cause hypertrophy [6-10]. However, the exact mechanism is poorly understood [11-13].

Succinate is an important intermediate metabolite of the citric acid cycle and in conditions linked with insufficient blood supply, such as ischemia, succinate blood levels may rise [14,15]. The formation of succinate during ischemia occurs in different ways: in the presence or in the absence of alpha-ketoglutarate (anaplerotic reaction) [16,17]. In the reaction in which alpha-ketoglutarate is present, the reactive oxygen species (ROS) that are increased during ischemia promote decarboxylation of alpha-ketoglutarate, resulting in a non-enzymatic formation of succinate that can occur both in the mitochondria and in the cytosol [17]. In the absence of alpha-ketoglutarate, the substrates that form succinate are especially amino acids glutamine and alanine. These amino acids provide one carbon skeleton for the formation of succinate [14,17-20].

Besides succinate crucial role in energy metabolism, it also acts as a signaling molecule by binding to and activating its specific G-protein coupled receptor (GPCR), known as GPR91 [21]. Signaling pathways triggered by GPR91 include increases in intracellular Ca^2+^ and cAMP, as well as activation of mitogen-activated protein kinases and extracellular signal–regulated kinases-1/2 (MAPK-ERK1/2) [21-23]. GPR91 was first reported in kidney [21], but more recently it was detected in other tissues, including cardiac muscle [15,21,22]. In cardiomyocytes, succinate modulates global Ca^2+^ transients and cell viability through a PKA-dependent pathway. In this study, we show for the first time that increased levels of succinate due to ischemia cause cardiac hypertrophy via GPR91 activation.

## Results

### Activation of GPR91 by succinate causes cardiomyocyte hypertrophy likely due to long-term blood pressure counter regulatory mechanisms

**Figure 1:**
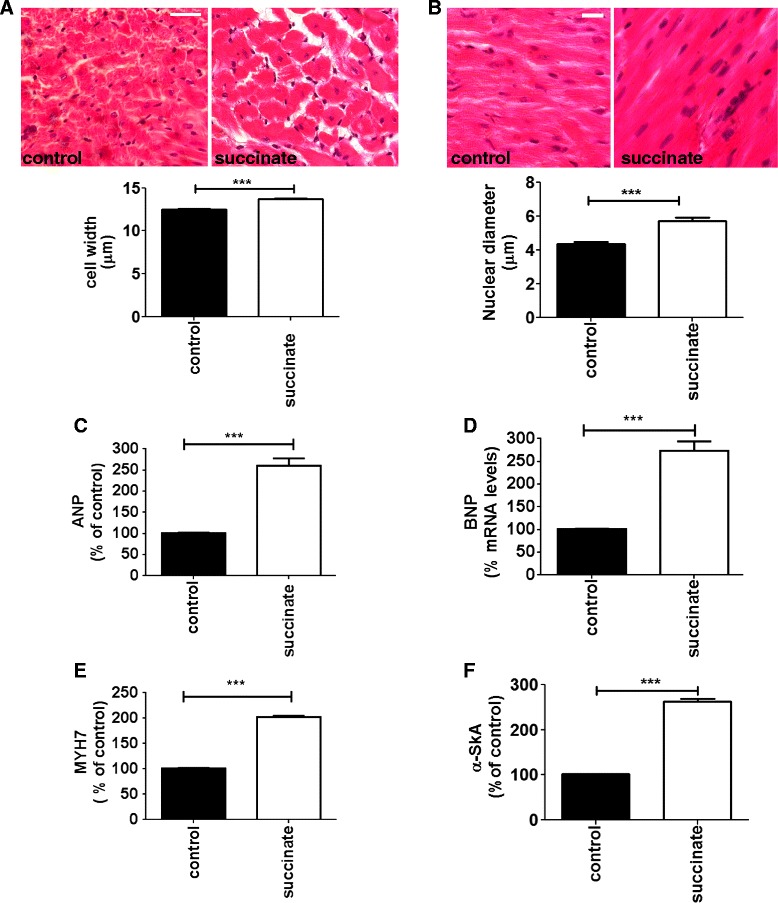
**Succinate induces pathologic cardiac hypertrophy. A**. Cardiac morphometric analysis after 5 days of consecutive succinate administration. Upper panels show representative images of cell width. The specimen was stained with hematoxylin and eosin (original magnification; x 100, scale bars; 50 μm). The bar graph shows quantitative analysis of cardiomyocyte diameter (n = 50, ***p < 0.001). **B**. The upper panels show representative images of the nuclear diameter. The bar graph summarizes data from the nuclear diameter. (n = 5, ***p < 0.001). Scale bar 6 μm. **C**. ANP mRNA levels in freshly isolated adult cardiomyocytes from control and succinate treated rats. **D**. BNP mRNA levels in freshly isolated adult cardiomyocytes from control and succinate treated rats. **E**. MYH7 mRNA levels in freshly isolated adult cardiomyocytes from control and succinate treated rats. **F**. α-SkA mRNA levels in freshly isolated adult cardiomyocytes from control and succinate treated rats. These results represent the mean ± S.E. of three separate experiments (***p < 0.001).

**Figure 2:**
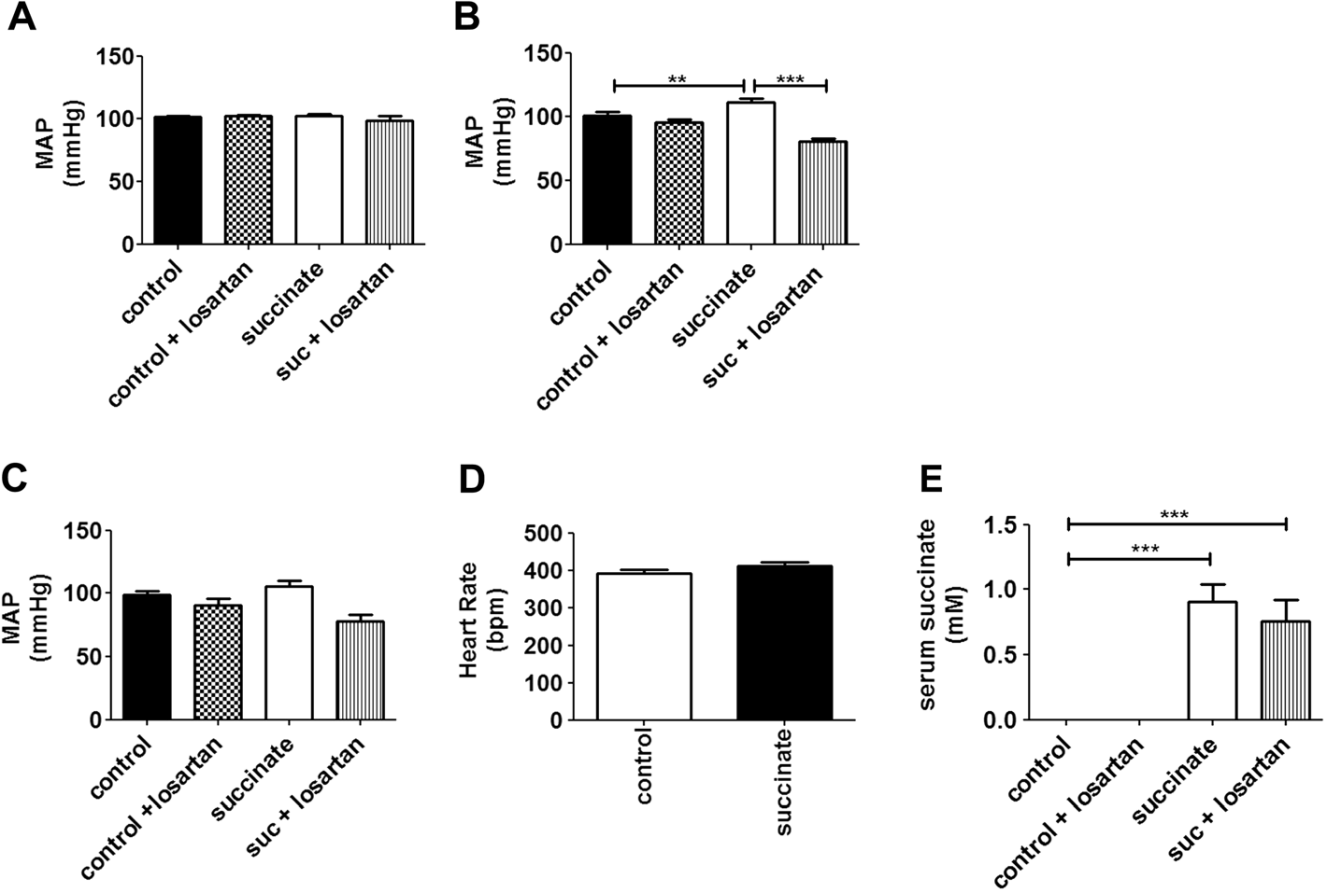
**Sustained levels of serum succinate cause increase in blood pressure on day 4 of treatment, but do not affect heart rate throughout the experiment.** Medium Arterial Pressure levels in control animals and succinate treated animals, with or without IV injection of losartan on the 3^rd^ day **(A)**, 4^th^ day **(B)** and 5^th^ day of treatment **(C)** (**p < 0.01, ***p < 0.001).** D**. Mean heart rate of control and succinate treated rats during the experiment. **E**. Serum succinate concentration measured on the last day of experiment.

**Figure 3:**
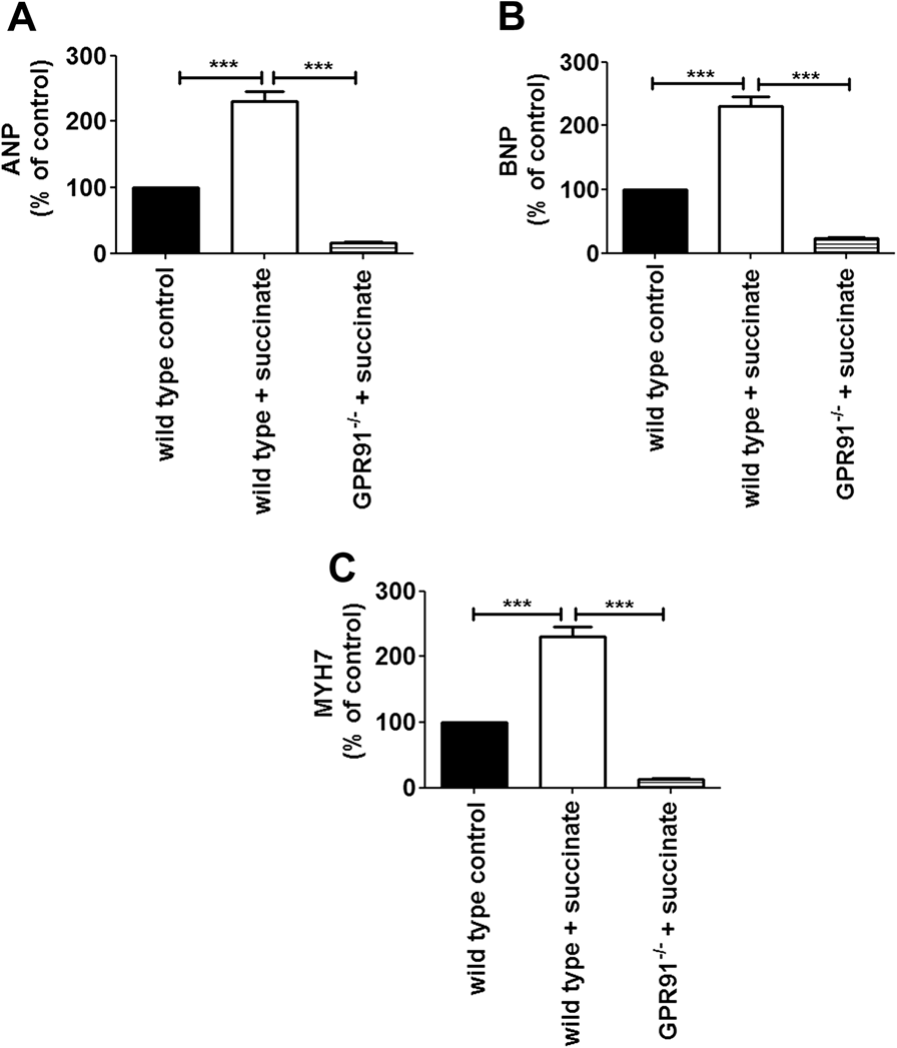
**High blood levels of succinate induce cardiac hypertrophy through GPR91. A-C**. ANP, BNP and BNP mRNA levels in cardiomyocytes from control (WT) and knockout mice (GPR91^−/−^) subjected to intravenous administration of succinate for 5 days (n = 6, ***p < 0.001).

Extracellular accumulation of up to millimolar levels of succinate is observed in pathophysiological conditions, such as ischemia [14,15,21]. Moreover, there is a close correlation between myocardial ischemia and hypertrophic cardiomyopathy [24,25]. To investigate whether succinate causes cardiac hypertrophy, a succinate dose of 0.066 mg/kg, equivalent to that observed during ischemic events [15], was administered intravenously to rats, once a day, for 5 consecutive days. By using perfused-fixed hearts from PBS (control) and succinate treated animals, we evaluated changes in cardiomyocyte width from left cardiac ventricle sections in longitudinal orientation. Morphometric analysis showed a significant increase in average myocyte width of groups treated with succinate compared to controls, (12 ± 0.10 μm in control *vs* 14.7 ± 0.10 μm in succinate treated animals, p < 0.001), (Figure 1A). We also observed a significant increase in myocyte nuclear diameter in succinate treated groups (4.3 ± 0.12 μm in cardiomyocytes from control animals *vs* 5.6 ± 0.2 μm in succinate treated rats, p < 0.001), (Figure 1B), suggesting that high circulating succinate levels might cause cardiac hypertrophy. This finding was confirmed by evaluating the expression levels of genes expressed during cardiac hypertrophy. We detected upregulation of hypertrophic markers atrial natriuretic peptide (ANP), brain natriuretic peptide (BNP), and β-myosin heavy chain (MYH7). Moreover we fond a significant increase in α-skeletal actin (α-SkA) mRNA levels, a known marker for pathological hypertrophy, in heart samples from succinate treated groups. We observed an increase of 160% in the expression level of ANP (a.u. = 100 in control cells vs. 260 ± 6.5% in cells from succinate treated rats, p < 0.001), an increase of 175% in the expression of BNP (a.u. = 100% in cells from control rats *vs* 275 ± 10% in cells from succinate treated rats, p < 0.001), an increase of 125% in the expression of MYH7 (a.u. = 100% in cells from control rats *vs* 225 ± 3% in cells from succinate treated rats*,* p < 0.001), and increase of 168% in the expression level of α-SkA (a.u. = 100 in control cells *vs* 268 ± 6.3% in cells from succinate treated rats, p < 0.001) in freshly isolated adult cardiomyocytes from succinate treated rats when compared to control animals (Figure 1C-F). Succinate is knows to activate the renin angiotensin system (RAS) [21], which can modulate blood pressure [26]. Since sustained increase in blood pressure is know to cause cardiac hypertrophy [27,28], we investigated whether the hypertrophy induced by high levels of succinate in the blood stream was a consequence of succinate triggering changes in the arterial blood pressure. Under our experimental conditions, we found that the mean arterial blood pressure (MAP) level was unaffected after two days of succinate treatment, but slightly increased at day 4, and reverted to normal values on the final day of the experiment (day 3: 101.9 ± 0.78 mmHg in control rats, 109 ± 0.42 mmHg in control rats treated with losartan, 102 ± 1.52 mmHg for succinate-treated rats and 99 ± 3.85 mmHg for rats treated with succinate and losartan, day 4: 101.2 ± 2.86 mmHg in control rats, 95.82 ± 1.88 mmHg in control rats treated with losartan, 111.6 ± 2.60 mmHg in succinate-treated rats and 79.92 ± 2.48 mmHg in rats treated with succinate and losartan, day 5: 98.8 ± 2.94 mmHg in control rats, 90.43 ± 5.2 mmHg in control rats treated with losartan,105.3 ± 4.8 mmHg in succinate treated rats, 77.51 ± 5.79 mmHg in rats treated with succinate and losartan). These variations occurred without any change on the heart rate (Figure 2A-D), even though at the last experimental day, the serum concentration of succinate was significantly higher in treated animals compared to control (0 mM in control rats *vs* 0.9 ± 0.13 mM in succinate treated rats, p < 0.001), (Figure 2E). We noticed that increases in blood pressure induced by succinate were reverted by losartan, a well-known inhibitor of type I angiotensin-II receptor [29]. Additionally, the consequences of *in vivo* succinate exposure for cardiac function were further investigated by echocardiography experiment in the presence or absence of losartan. Table 1 shows that succinate increased cardiac output (52.17 ± 5 in control rats *vs* 68.03 ± 2.9 in succinate rats, p < 0.05), left ventricular end diastolic volume (LVd: 180 ± 9.7 in control rats *vs* 245.20 ± 10.11 in succinate rats, p < 0.01), stroke volume (SV: 136.7 ± 12.63 in control rats *vs* 167.5 ± 7.34 in succinate rats, p < 0.01), and left ventricular chamber dimension, at both systole (LVIDs: 3.02 ± 0.06 in control rats *vs* 3.96 ± 0.10, p < 0.01) and diastole (LVIDd: 6.15 ± 0.22 in control rats *vs* 6.99 ± 0.09, p < 0.01). Although losartan did not affect most evaluated parameters, it slightly attenuated succinate-induced increase in left ventricular chamber diameter during diastole (LVIDd: 6.99 ± 0.09 in succinate rats *vs* 6.50 ± 0.15 in succinate + losartan, p < 0.05). Real time PCR of hypertrophic markers from these experimental groups showed that losartan partially reverted the re-expression of ANP and MYH7 induced by succinate. We observed a decreased of 110.7% in the expression level of ANP (a.u. = 304 ± 4.33% in rats hearts treated with succinate vs. 193.3 ± 6.67% in rats hearts treated with succinate in the presence of losartan, p < 0.001), and a decreased of 38.7% in the expression of MYH7 (a.u. = 174 ± 3.05% in rats hearts treated with succinate *vs* 135.3 ± 2.9% in in rats hearts treated with succinate in the presence of losartan, p < 0.001), (Additional file 1: Figure S1A-B). Thus, these findings corroborate previous data showing that succinate activates RAS [21,23,30]. To address the role of GPR91 on succinate-induced cardiac hypertrophy, we performed echocardiogram using GPR91 knockout (GPR91 KO) mice. Table 2 shows that GPR91-KO mice have no alteration in echocardiographic parameters when compared to wild type. Despite the absence of GPR91, KO mice treated with succinate showed changes in left ventricular ejection fraction (LV-EF 44.33 ± 0.35 in wild mice *vs* 57.37 ± 0.77 in wild mice treated with succinate, p < 0.05, and 38.17 ± 1.4 in GPR91 KO mice *vs* 52.9 ± 4.7 in GPR91 knockout mice treated with succinate, p < 0.05), fractional shortening (LV-FS: 21.51 ± 0.23 in wild mice *vs* 29.86 ± 0.23 in wild mice treated with succinate, p < 0.05, and 18.05 ± 0.70 in GPR91 knockout mice *vs* 26.14 ± 2.97 in GPR91 knockout mice treated with succinate, p < 0.05), and systolic volume (LV-SV: 32.65 ± 0.65 in wild mice *vs* 22.13 ± 0.87 in wild mice treated with succinate, p < 0.01, and 31.43 ± 3.44 in GPR91 knockout mice *vs* 17.76 ± 1.36 in GPR91 knockout mice treated with succinate, p < 0.01)*.* Importantly, succinate-induced increase in left ventricular posterior wall (LVPWs: 0.64 ± 0.09 in wild mice *vs* 1.05 ± 0.07 in wild mice treated with succinate, p < 0.05) was observed only in wild type mice, indicating that GPR91 is essential for succinate-induced hypertrophic effects on the heart. We also evaluated the expression levels of the aforementioned hypertrophic markers in cardiomyocytes isolated from GPR91 deficient animals. We found that in the absence of GPR91, injection of succinate was unable to induce the expression of ANP (a.u. = 100% in cells from WT mice *vs* 230 ± 15% in cells from succinate treated WT mice *vs* 15 ± 2% in cells from GPR91^−/−^ mice treated with succinate *vs* 120% in cells from GPR91^−/−^, p < 0.001), BNP (a.u. = 100% in cells from WT mice *vs* 230 ± 15% in cells from succinate treated WT mice *vs* 23 ± 1% in cells from GPR91^−/−^ mice treated with succinate *vs* 116 ± 2% in cells from GPR91^−/−^, p < 0.001), and MYH7 (a.u. = 100% in cells from WT mice *vs* 230 ± 15% in cells from succinate treated WT mice *vs* 12 ± 1.4% in cells from GPR91^−/−^ mice treated with succinate, *vs* 108 ± 4% in cells from GPR91^−/−^*,* p < 0.001), (Figure 3A-C). The involvement of other key TCA cycle intermediate was not considered here since it was already demonstrated that succinate is the only agonist for GPR91 [21]. Taken together, these results show that high circulating levels of succinate lead to cardiac hypertrophy, through direct activation of GPR91. Nonetheless, the results also suggest that succinate-induced remodeling is not limited to its direct effects on the GPR91 in cardiac tissue, but might as well have its origin point in other organs besides the heart.

**Table 1:** **Echocardiographic parameters**

**Parameter**	**Control (n = 6)**	**Control + Losartan (n = 3)**	**Succinate (n = 8)**	**Suc + Losartan (n = 5)**
**Cardiac output (mL/min)**	52.17 ± 5.029	52.67 ± 4.7	68.03 ± 2.9*	56.14 ± 3.12
**LV ejection fraction (%)**	71.74 ± 3.02	75.43 ± 1.78	74.00 ± 1	71.76 ± 1.00
**LV fraction shortening (%)**	40.95 ± 2.57	45.20 ± 1.81	43.42 ± 0.9	41.87 ± 0.87
**LVd (μL)**	180.00 ± 9.7	203.90 ± 8.5	245.20 ± 10.11^#^	209.30 ± 12.09
**LV systolic volume (μL)**	72.98 ± 6.34	44.64 ± 7.31	62.70 ± 7.53	57.06 ± 3.9
**Stroke volume (μl)**	136.70 ± 12.63	137.20 ± 12.99	167.50 ± 7.34^#^	142.10 ± 6.9
**Heart rate (bpm)**	398.00 ± 9.2	399.40 ± 10.19	402.90 ± 7.9	393.60 ± 17.18
**LVIDd (mm)**	6.15 ± 0.22	6.32 ± 0.11	6.99 ± 0.09^#^	6.50 ± 0.15^&^
**LVIDs (mm)**	3.02 ± 0.06	3.49 ± 0.14	3.96 ± 0.10^#^	3.70 ± 0.10*

*p < 0.05 versus control.

^&^p < 0.05 versus succinate.

^#^p < 0.01 versus control.

**Table 2:** **Echocardiographic parameters**

**Parameter**	**Control (n = 3)**	**Succinate (n = 3)**	**GPR91** ^**−/−**^ **(n = 4)**	**GPR91** ^**−/−**^ **+ suc (n =3)**
**Cardiac output (mL/min)**	13.09 ± 1.19	13.02 ± 0.46	10.64 ± 0.55	9.18 ± 1.29
**LV ejection fraction (%)**	44.33 ± 0.35	57.37 ± 0.77*	38.17 ± 1.4	52.9 ± 4.7^&^
**LV fraction shortening (%)**	21.51 ± 0.23	29.86 ± 0.23*	18.05 ± 0,70	26.14 ± 2.97^&^
**Interventricular septal**				
**Dimension (diastole, mm)**	0.56 ± 0.02	0.54 ± 0.03	0.57 ± 0.05	0.58 ± 0.01
**LVd (μL)**	3.71 ± 0.03	3.54 ± 0.04	3.72 ± 0.1	3.25 ± 0.12^&^
**LV posterior wall (systole, mm)**	0.64 ± 0.09	1.05 ± 0.07*	0.80 ± 0.02	0.75 ± 0.12
**LV systolic volume (μL)**	32.65 ± 0.65	22.13 ± 0.87^#^	31.43 ± 3.44	17.76 ± 1.36^##^
**Stroke volume (μl)**	25.42 ± 1.01	30.56 ± 0.79	22.88 ± 1.22	20.67 ± 2.41
**Heart rate (bpm)**	501.2 ± 31	426.1 ± 13	470.7 ± 11	434.6 ± 11.6

Echocardiographic measurements of cardiac parameters in mice following succinate intravenous injection for 5 days.

* < 0.05 versus control.

^&^p < 0.05 versus GPR91 ^−/−^.

^#^p < 0.01 versus control.

^##^p < 0.01 versus GPR91 ^−/−^.

### Succinate-induced cardiomyocyte hypertrophy involves activation of MEK/ERK1/2 and HDAC5 pathways

**Figure 4:**
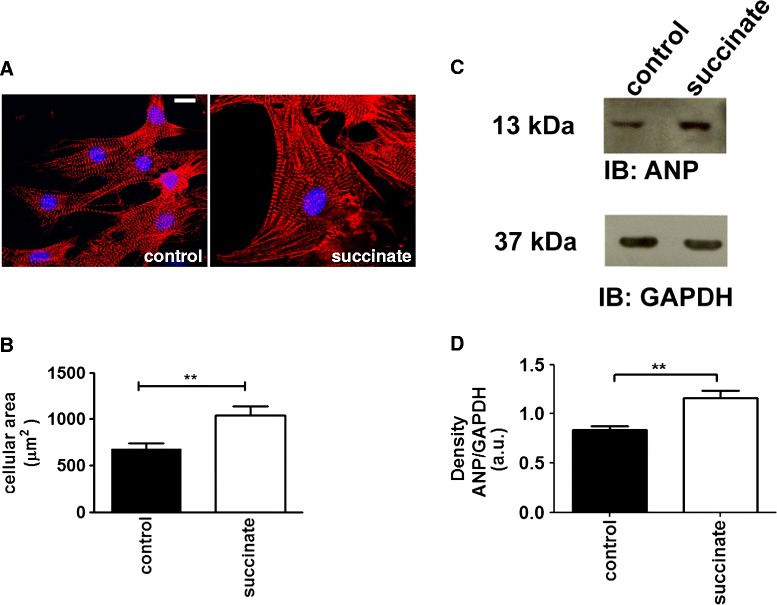
**Succinate induces hypertrophy in neonatal cardiomyocytes. A**. Representative confocal images of neonatal cardiomyocytes double-labeled with DAPI (blue), and anti-α-actinin (red). Left panel shows control cells and right panel shows cells treated with succinate. **B**. Summary of cellular area, indicating that succinate induces hypertrophy. (*p < 0.05, n = 35 cells). **C**. Representative immunoblot of whole-cell protein lysates from neonatal cardiomyocytes probed with anti-ANP and anti-GAPDH. **D**. Bar graph shows that succinate significantly increases ANP levels. These results represent the mean ± S.E. of three separate experiments (**p < 0.01).

**Figure 5:**
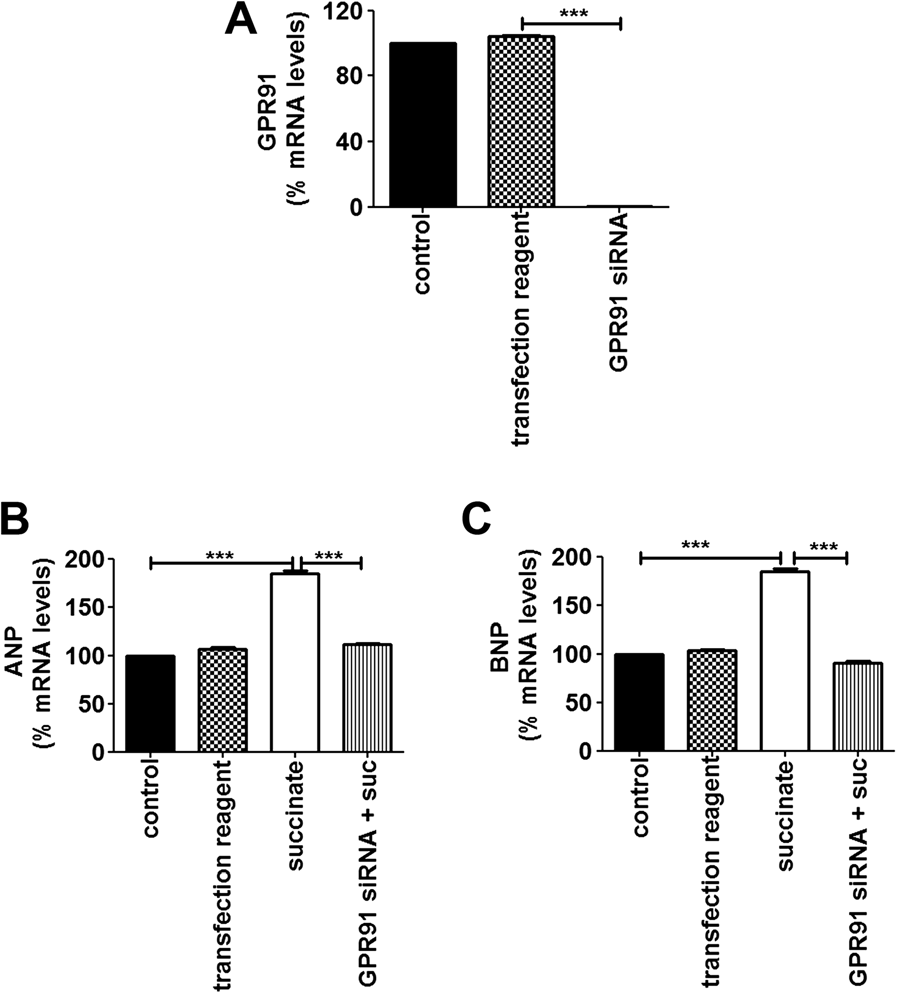
**Hypertrophic effect of succinate in cardiomyocytes requires GPR91 expression. A**. Real time PCR showed significant knockdown of GPR91 mRNA levels in cells transfected with siRNA against GPR91 (n = 3 independent experiments, ***p < 0.001). **B-C**. Knockdown of GPR91 decreased ANP and BNP mRNA levels. (***p < 0.001).

GPR91 can be coupled with different second messenger signaling systems, depending on the individual cell type where it is expressed. For instance, succinate uses both G_i_/G_o_ and Gq/11 pathways to increase ERK1/2 phosphorylation and intracellular Ca^2+^ [21-23,31], which are, in fact, pathways involved in cardiac hypertrophy induced by G-protein coupled receptors [5,13,32,33]. In order to gain further insight into the mechanisms by which succinate induces cardiomyocyte hypertrophy and the dependence of this process on GPR91, we used primary cultures of neonatal rat cardiomyocytes. We first investigated whether *in vitro* succinate treatment could also induce hypertrophy. For that, we tested several different concentrations of succinate, and measured cellular width after treatment with each of these concentrations (500 ± 12 μm^2^ for control cells; 682 ± 20 μm^2^ for cells treated with 25 mM succinate; 800 ± 15 μm^2^ for 0.5 mM succinate, 1000 ± 15 μm^2^ for 0.75 mM succinate, 1041 ± 25 μm^2^ for 1 mM succinate, 1083 ± 13 μm^2^ for 1.5 mM succinate, 1080 ± 10 μm^2^ for 2 mM succinate, 1085 ± 20 μm^2^ for 2.5 mM succinate, Additional file 2: Figure S2). We found that neonatal cardiomyocytes exposed to 1 mmol/L succinate for 36 hours had increased cell surface area when compared to controls (600 μm^2^ ± 15 in cells from control *vs* 1100 μm^2^ ± 20 in cells from succinate treated cardiomyocytes, p < 0.01, n = 60 cells), (Figure 4A-B). In addition, succinate treatment led to an increase in ANP expression levels (a.u. = 0.93 ± 0.06 in cells from control *vs* 1.3 ± 0.10 in cells from succinate treated cardiomyocytes, n = 3 independent experiments, p < 0.01), (Figure 4C-D), providing evidence for onset cardiomyocyte hypertrophy, an effect that was not observed upon knockdown of succinate receptor with GPR91 siRNA. Real time PCR analysis indicated that siRNA against GPR91 abolished the expression of succinate receptor (Figure 5A) and prevented the increase of hypertrophic markers induced by succinate: ANP (a.u. = 100% in control cells *vs* 180 ± 8% in succinate treated cells *vs* 100 ± 1.4% in cells with GPR91siRNA, p < 0.001) and BNP (a.u. = 100% in control cells *vs* 180 ± 8% in succinate treated cells *vs* 95 ± 1.4% in cells with GPR91siRNA, n = 3 independent experiments, p < 0.001), (Figure 5B-C). These findings show that succinate causes hypertrophy due to a direct activation of GPR91 in cardiomyocytes.

**Figure 6:**
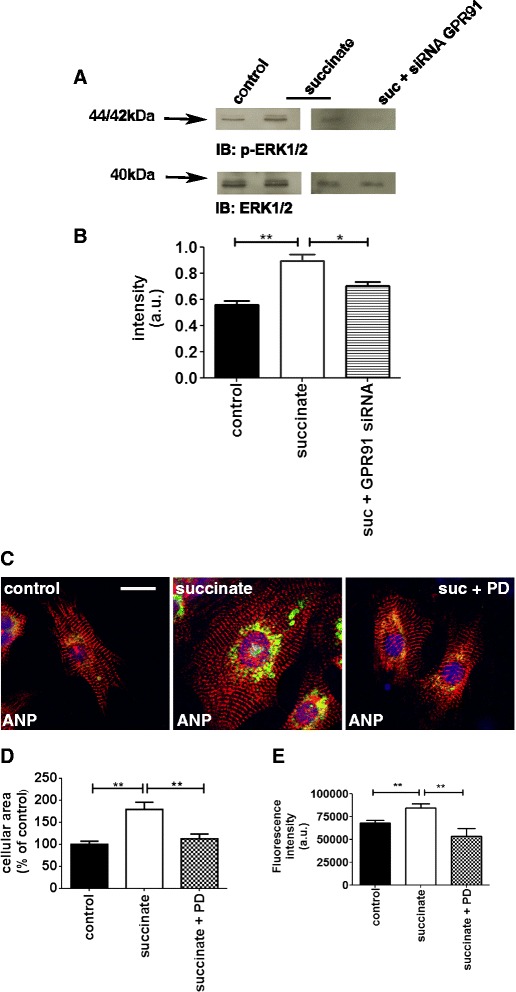
**Succinate activates the ERK1/2 hypertrophic signaling pathway. A**. Immunoblot of whole cell lysates showing increased phosphorylation of ERK1/2 and absence of phosphorylated ERK1/2 when GPR91 is silenced with siRNA. **B**. Bar graph shows that succinate significantly increases phosphorylation of ERK1/2 levels and fails to increase ERK1/2 phosphorylation when GPR91 is efficiently silenced. These results represent the mean ± S.E. of three separate experiments (**p < 0.01). **C**. Cells were treated with succinate and ERK1/2 inhibitor PD 098059. Immunofluorescence staining with DAPI (blue), anti-α-actinin (red) and ANP (green). **D-E**. Summary of cellular area and fluorescence intensity indicating that inhibition of ERK1/2 signaling pathway reverses the hypertrophic effect of succinate. (**p < 0.01, n = 50 cells).

Because ERK1/2 phosphorylation is a classical MAPK pathway activated in cardiomyocyte hypertrophy [13,32], and GPR91 activation is known to be associated with the ERK1/2 signaling cascade [21,23,31], we investigated whether succinate treatment alters ERK1/2 phosphorylation levels in cardiomyocytes as well. We found that exposing cardiomyocytes to succinate increased phosphorylation of ERK1/2, without affecting its expression levels (a.u. = 0.58 ± 0.02 in control cardiomyocytes *vs* 0.78 ± 0.02 in succinate treated cardiomyocytes, p < 0.01, n = 3 independent experiments). We also observed that efficient silencing of GPR91 prevented the increase in ERK1/2 phosphorylation upon succinate treatment (a.u. = 0.58 ± 0.02 in control cardiomyocytes *vs* 0.89 ± 0.05 in succinate treated cardiomyocytes *vs* 0.70 ± 0.03 in succinate treated cardiomyocytes transfected with GPR91siRNA, p < 0.01, n = 3 independent experiments), (Figure 6A-B). Moreover, the effects of succinate on cellular area and ANP expression were prevented by PD098059, an inhibitor of MEK1/2, (a.u. = 100% in control cells *vs* 180 ± 12% in cells treated with succinate *vs* 125 ± 5% in cells treated with succinate in the presence of PD098059, *vs* 109 ± 4% in PD098059 treated cells alone; (fluorescence intensity: a.u. = 62500 ± 500 in control cells *vs* 87500 ± 763 in cells treated with succinate *vs* 50500 ± 10000 in cells treated with succinate in the presence of PD098059, *vs* 71000 ± 577% in PD098059 treated cells alone; n = 45, p < 0.01), (Figure 6C-E). We observed a very low expression of ANP, close to the control level. Since PD098059 is specific to MAPK inhibition, this data may suggest the existence of another protein, downstream to the MAPK inhibition, that negatively feedbacks the pathway, inactivating other important intermediate or adding to MEK inhibition, contributing to the lower activity caused by PD098059 [34-36]. However, altogether these findings show that MEK/ERK cascade is a crucial downstream pathway of the hypertrophic effects induced by succinate.

**Figure 7:**
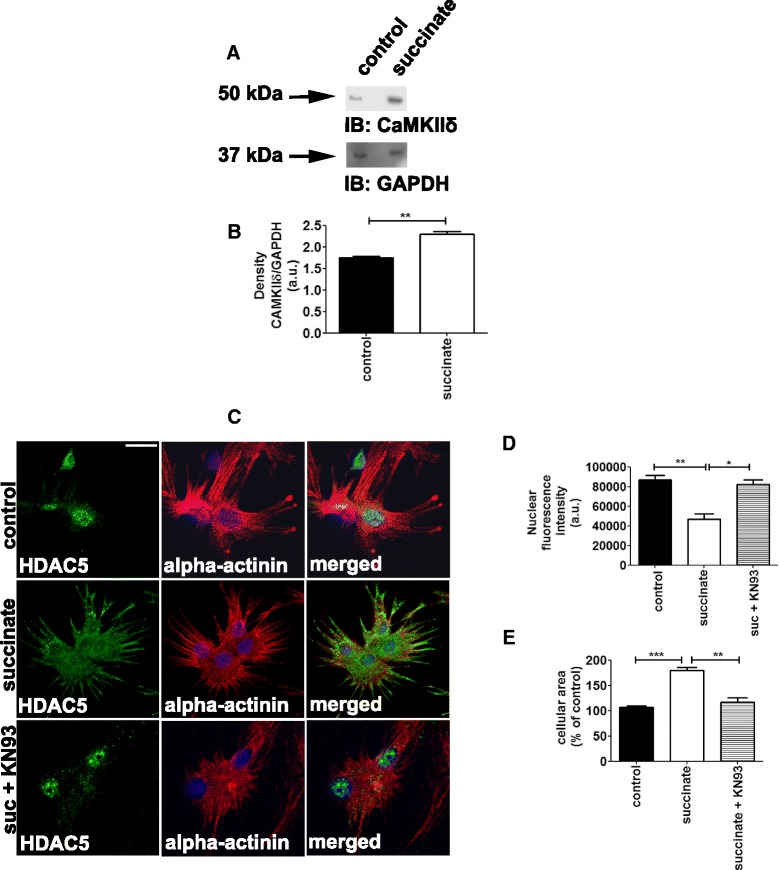
**Succinate activates the CaMKII**δ **hypertrophic signaling pathway. A**. Immunoblot for CaMKIIδ of whole cell lysates from primary cultures of neonatal cardiomyocytes. **B**. Bar graph shows that succinate significantly increases CAMKIIδ levels. These results represent the mean ± S.E. of three separate experiments. (**p < 0.01). **C**. Representative images of cardiomyocytes immunostained with antibodies against HDAC5 (green), α-actinin (red) and DAPI (blue). Succinate decreased HDAC5 nuclear export. Scale bar represents 10 μm. KN93 is a selective CaMKIIδ inhibitor. Nuclear export of HDAC5 induced by succinate is dependent on CaMKIIδ.** D**. Quantification of the nuclear fluorescence for HDAC5 (*p < 0.05, **p < 0.01). **E**. Summary of cellular area (**p < 0.01, ***p < 0.001), 45 cells).

Additional important signaling cascades that are well-known to be involved in cardiomyocyte hypertrophy are the calcineurin-NFAT [2,3] and the histone deacetylase (HDAC) [37] pathways, both activated upon an increase in intracellular Ca^2+^ signals [38]. We have previously demonstrated that succinate increases intracellular Ca^2+^ transients in cardiomyocytes [22]. Thus, we now investigated whether exposing cardiomyocytes to succinate would trigger the above-mentioned Ca^2+^-dependent hypertrophic signaling cascades. We found that succinate did not activate NFAT, represented by the absence of NFAT in the nucleus upon succinate treatment (Additional file 3: Figure S3A-B). Instead, succinate activated calmodulin kinase IIδ (CaMIIδ), (a.u. = 1.75 ± 0.06 in control cells, *vs* 2.30 ± 0.1 in succinate treated cells, n = 3, p < 0.01), (Figure 7A-B), and HDAC5 signaling pathway (Figure 7C-E). HDAC5 translocated from the nucleus to the cytosol upon succinate treatment, an effect that was prevented by KN93, an inhibitor of CaMKIIδ [39], (a.u. = 93000 ± 1528% in control cells, *vs* 46000 ± 3000% in succinate treated cells, *vs* 82000 ± 9000% in succinate and KN93 treated cells, *vs* 93000 ± 2000% in KN93 treated cells alone, p < 0.001, p < 0.01), (Figure 7C-D**)**. The increase in cellular area induced by succinate was also prevented by KN93 treatment (a.u. = 100 ± 3.68% in control cells, *vs* 180 ± 5.45% in succinate treated cells, *vs* 117 ± 8.35% in succinate and KN93 treated cells, *vs* 114 ± 0.8% in KN93 treated cells alone, p < 0.001, p < 0.01), (Figure 7E). Part of KN93 effect on cellular area might be due to its unspecific effect as an antagonist. Validating this signaling pathway we further found that efficient silencing of GPR91 prevented both the translocation of HDAC5 from the nucleus to the cytosol (a.u. = 91667 ± 4410 in control cells, *vs* 88333 ± 4410 in GPR91 siRNA cells, *vs* 50667 ± 3480 in succinate treated cells, *vs* 89667 ± 333.3 in GPR91 siRNA and succinate treated cells, p < 0.001), and the increase in cellular area induced by succinate (a.u. = 102 ± 0.57% in control cells, *vs* 84 ± 0.57% in GPR91 siRNA cells, *vs* 156 ± 2% in succinate treated cells, *vs* 103.3 ± 8.8% in GPR91 siRNA and succinate treated cells, p < 0.001), (Additional file 4: Figure S4A-C). These results indicate that succinate causes cardiomyocyte hypertrophy through the activation of CaMKIIδ/HDAC5 pathway.

To verify whether succinate triggers similar hypertrophic signaling cascades *in vivo* as well, we intravenously administered 0.066 mg/kg succinate in 8-week-old rats. Succinate was injected for 5 days, once a day, and the expression pattern of phospho-ERK1/2 and changes in intracellular Ca^2+^ transients were evaluated in freshly isolated adult cardiomyocytes. We found that high circulating levels of succinate increased the expression of phospho-ERK1/2 compared to control cells (a.u. = 1 ± 0.3 in cells from control rats *vs* 2 ± 0.25 in cells from succinate treated cardiomyocytes, p < 0.01, n = 3), (Additional file 5: Figure S5A-B). We also found that administration of succinate for 5 consecutive days modulated Ca^2+^ transients in isolated ventricular myocytes loaded with the Ca^2+^ fluorescent probe fluo-4/AM. As expected, succinate increased the amplitude (3.8 ± 0.4 F/F_0_ in cells from control rats *vs* 5.2 ± 0.6 F/F_0_ in cells from succinate treated cardiomyocytes, p < 0.01, n = 40 cells), and decreased the decay rate of the Ca^2+^ transient, (950 ± 9 ms in cells from control rats *vs* 920 ± 7 ms in cells from succinate treated cardiomyocytes, p < 0.05, n = 40), (Additional file 6: Figure S6A-D). Collectively, these results show that succinate activates intracellular hypertrophic signaling cascades, both *in vitro* and *in vivo*.

### Ischemic diseases increase succinate blood level in patients

Ischemia is known to raise plasma succinate to millimolar levels in rodents [40]. We now used HPLC to evaluate the concentration of circulating succinate in human serum. For that, we first used serum samples of rodents previously subjected to ischemia-reperfusion procedure, to standardize the method (p < 0.001, Additional file 7: Figure S7). We then investigated whether patients with hypertrophic cardiomyopathy associated with ischemia had altered levels of succinate in their blood flow. Nine patients diagnosed with acute myocardial infarction and/or chronic coronary artery disease showed high blood concentration of succinate, (0.9 ± 0.1 mmol/L of succinate in serum of patients with coronary artery disease *vs* 1 ± 0.2 mmol/L of succinate in patients with acute myocardial infarction *vs* 2.69 mmol/L of succinate in patients with myocardial infarction plus coronary artery disease), compared with 6 control subjects of similar age, with undetectable serum levels of succinate (Table 3). Moreover, we found elevated levels of NT–pro–BNP in all subjects with ischemic disease, although higher levels of NT–pro–BNP were detected in patients with chronic coronary artery disease in comparison with patients with acute myocardial infarction, (coronary artery disease: 8101 ± 4524 pg/mL *vs* 171 ± 101 pg/mL acute myocardial infarction). To verify whether other more severe acute ischemic conditions could also cause changes in succinate blood levels, we measured succinate concentration in the serum of patients that underwent hepatic transplantation. In these patients, we evaluated the concentration of succinate 1 hour and 6 hours post transplantation (Table 4). We found that ischemia-reperfusion injury elevated succinate levels in a time dependent manner (1.90 ± 0.2 mmol/L of succinate 1 hours post transplantation and 2.36 ± 0.2 mmol/L of succinate 6 hours post transplantation). More information about the patients can be seen elsewhere (Additional file 8: Table S1). Although preliminary, these data suggest that succinic acid accumulation in the blood is a possible marker to indicate the presence of ischemia and a potential target to prevent further damage, such as cardiomyocyte hypertrophy.

**Table 3:** **Characteristics of the control patients and cardiac and plasma levels of succinate**

**Study characteristics**	
Number patients, n	15
Control	6
Cardiac	9
Median age, years	62 ± 2.7*
**Pathological condition**	Number of patients
Coronary artery disease, n	5
Acute myocardial infarction, n	3
Coronary artery disease + Acute myocardial infarction, n	1
Total	9
**Median blood levels NT-pro-BNP (pg/mL)**	
Coronary artery disease	8101 ± 4524*
Acute myocardial infarction	171 ± 101*
Coronary artery disease + Acute myocardial infarction	25000
**Median blood levels succinate (mmol/L)**	
Coronary artery disease	0.9 ± 0.1*
Acute myocardial infarction	1 ± 0.2*
Coronary artery disease + Acute myocardial infarction	2.69

*The results are expressed as mean ± S.E.M.

**Table 4:** **Patients characteristics and serum levels of succinate post- hepatic transplantation**

**Study characteristics**	
Number patients, n	8
Median age, years	46 ± 7*
**Pathological condition**	Number of patients
Cirrhosis due to hepatitis C	4
Cirrhosis due to Wilson disease	2
Cirrhosis due to alchohol and hepatocellular carcinoma	1
Cirrhosis due hepatitis C and hepatocellular carcinoma	1
Total	8
**Median blood levels succinate (mmol/L)**	Median (mmol/L)
1 hour post transplantation	1.90 ± 0.2*
6 hours post transplantation	2.36 ± 0.3*

*The results are expressed as mean ± S.E.M.

## Discussion

Cardiomyocyte hypertrophy is among the most common causes of ischemic heart disease and may result in myocardial infarction, thus promoting further ischemia. Even though this complex syndrome has been extensively investigated, there are still many aspects that remain elusive. In this work, using both human and rodent systems, we propose that succinate plays a key role in pathological cardiomyocyte hypertrophy. Moreover, our results shed some light on the mechanisms by which succinate, through its specific receptor GPR91 activation, induces hypertrophic cardiomyopathy.

Hypertrophic stimuli are mediated by several intracellular signaling cascades that ultimately cause reactivation of fetal cardiac genes involved in hypertrophy [41,42]. A central signaling cascade that has been implicated in the development of cardiac hypertrophy is the mitogen activated protein kinase (MAPK) cascade consisting of the kinases rapid activation of fibrosarcoma (Raf), MAP/ERK kinase (MEK1/2), and ERK1/2 [43]. HDAC is another well-established effector in the transmission of cardiac stress to hypertrophic gene expression. Here we show that activation of GPR91 by succinate causes phosphorylation of ERK1/2, as already demonstrated in other cell types [21,23,44]. Additionally, we found that succinate increased intracellular Ca^2+^ transients required for activation of CaMKIIδ and consequent HDAC5 phosphorylation. The current findings confirm previous data [21-23,31] and extend them by now showing for the first time that succinate *in vivo* also promotes increases in intracellular Ca^2+^ transient in ventricular myocytes. Moreover, our results are in agreement with previous reports that indicate CaMKIIδ as the main pathway involved in pathological hypertrophy [45,46]. Although ERK1/2 pathway is also known to induce cardiac hypertrophy, in part by activating a crosstalk with the calcineurin-NFAT circuit [47], we found that under our experimental conditions, succinate did not induce translocation of NFAT to the nucleus to activate pro-phypertrophic gene expression. It is known that calcineurin-NFAT signaling is controlled by other kinases that can directly phosphorylate the N-terminal regulatory domain of NFAT, antagonizing its nuclear occupancy [48-50]. Therefore, we cannot rule out that succinate could activate such kinases in cardiomyocytes, including JNK, GSK3, and p38. Indeed, activation of p38 pathway by succinate has been demonstrated in other cell types [30]. In cardiomyocytes, recent study demonstrated that succinate could activate PI3K/Akt signaling cascade [51], which is another pathway also implicated in cardiac hypertrophy [52].

Intravenous administration of succinate increases plasma renin activity and causes a dose-dependent increase in blood pressure, that could be blocked by angiotensin-converting enzyme inhibitors [21,23]. Moreover, high circulating succinate concentration was detected in spontaneously hypertensive rats [40]. Under our experimental conditions, we found that the mean arterial blood pressure was high after 4 consecutive days of succinate treatment, and shifted back to normal values at the final day of the experiment. We have not investigated the reason for such an oscillatory pattern of blood pressure, but it is possible that the succinate-treated animals experienced a type of compensatory response due to the abnormal succinate exposure. Indeed, blood pressure adaptation to hormone stimulation is a natural process, and has been broadly described [53]. Corroborating previous data, we also found that succinate effects on cardiac functions were partially reverted by an angiotensin-II receptor antagonist losartan. Losartan prevented the increase in stroke volume and cardiac output triggered by succinate, confirming that, at a systemic level, RAS activation plays a role in succinate-induced change in blood pressure. We also discovered that losartan partially prevented the expression of the hypertrophic fetal genes induced by succinate, indicating that, at least in part, the observed succinate-induced cardiac remodeling could be a consequence of changes in blood pressure due to RAS activation. Despite the fact, that we have shown a significant increase in cardiomyocyte diameter by morphometric studies, we found no alteration in LVP wall thickness by echocardiography studies and no fibrosis in cardiac specimens. Considering these findings, we believe that we are observing early stages of cardiac hypertrophy in 5-day succinate treated rats, and we speculate that longer exposure to succinate will be necessary in order to observe substantial pathological indicators of cardiac hypertrophy. On the other hand, similar 5-day succinate injection protocol caused significant increase in cardiac hypertrophy in mice. These differences in the susceptibility to cardiac hypertrophy, by *iv* administration of succinate, could be due to specie-sensitivity. If directly accessing the effect of succinate in primary culture of cardiomyocytes, a pathological type of hypertrophy is clearly demonstrated, by CaMKIIδ activation, as well as by α − SKA gene reexpression. Nevertheless, both with *in vivo* and *in vitro* studies, our data support a direct role of GPR91 in succinate-induced cardiac hypertrophy, since GPR91 ablation prevented: succinate-induced cardiac hypertrophy in GPR91KO mice, expression of hypertrophic markers, ERK1/2 activation, and intracellular HDAC5 translocation. Even though succinate can induce cardiac hypertrophy – through systemic augmentation of renin-angiotensin II activity and local activation of the above mentioned hypertrophic cellular signaling cascades in cardiomyocytes – GPR91 is a converting point in both of them. We still cannot rule out the possibility that succinate might bind to other not yet characterized receptor(s), which may activate distinct intracellular signaling pathway to differently regulate cell function.

Myocardial ischemia is accompanied by a variety of metabolic alterations in myocardial tissue. Succinic acid, for instance, was observed to accumulate in severely as well as in moderately ischemic rabbit hearts, with a good correlation between degree plus duration of myocardial ischemia and tissue succinate content [54]. Using HPLC measurements, we now show that upon ischemic injury, succinate concentration also reaches blood flow. We found that succinate concentration increases in the serum of patients with cardiac hypertrophy associated with acute or chronic obstructive coronary artery diseases. Whether succinate was among the causes of the cardiac hypertrophy observed in these patients, or a consequence of the ischemic process, or both, still remain to be determined. However, our data show that succinate induces cardiomyocyte hypertrophy in cardiac cells though direct GPR91 activation. Thus, the presence of high circulating levels of succinate in the blood flow might reinforce the already installed hypertrophic phenotype that can lead to myocardial infarction, one of the ultimate consequences of ischemic heart disease.

High succinate concentrations have been detected in several other scenarios such as in blood samples from rodent models of hypertension and metabolic disease (type 2 diabetes) [40,55], in clinical specimens of patients with peritonitis [55], as well as in perfusate of rat liver under hepatic ischemia [15]. In line with these reports, we found that succinate is also increased in serum of patients that underwent liver transplant, a process that involves a transitory ischemic condition. Indeed cardiovascular diseases make up the most common cause of death in patients with functioning allografts at all times after transplantation, accounting for 30% mortality overall, with highest rates in the peritransplantation period. Therefore, GPR91 antagonism in preservation solution for transplantation could represent, for instance, a real benefit to help preventing cardiac hypertrophy due to organ transplant. Moreover, during transplant, succinate has been pointed as an ‘alarming’ signal able to trigger GPR91 to sense immunological danger and to increase allograft rejection [56]. In fact, a multiorgan failure has been reported in a liver-intestine transplant from a pediatric donor with a succinate- cytochrome C-reductase deficiency, a condition that raises succinate blood levels [57]. It has also been demonstrated that a patient with succinate dehydrogenase deficiency, another condition that also leads to extracellular accumulation of succinate, exhibited congestive heart failure [58]. Thus, succinate might be a clinical marker for ischemia, and its increase at blood level during organ transplant should be prevented, since it may cause, among other damages, cardiac hypertrophy.

## Conclusions

Collectively, our data strengthen previous results showing that succinate can be found outside the cell during ischemia and act as a circulating hormone. Our results also expand previous findings by showing that succinate, through GPR91 activation, induces cardiac hypertrophy. Interfering with this mechanism might prove as a powerful strategy in the prevention of cardiac hypertrophy and consequently heart failure.

## Methods

### Materials and reagents

Cardiomyocyte primary culture extracting kit was obtained from Worthington Biochemical Corporation (Lakewood, USA). Dulbeccos’s Modified Eagle’s Medium (DMEM), penicillin, streptomycin, amphotericin and fetal bovine serum (FBS) were purchased from Gibco (Grand Island, USA). Cytosine β-D-arabinofuranoside (ARA-C), fibronectin, KN-93 (Ca^2+^/calmodulin-dependent protein kinase II inhibitor) and ultra-pure succinic acid were obtained from Sigma (St. Louis, USA), Fluo-4/AM, DAPI and secondary antibodies conjugated to Alexa-488, Alexa-633 and Silencer kit were from Ambion, Life Technologies (New York, USA). Trizol reagent was obtained from Invitrogen (Eugene, USA). Polyclonal anti- GAPDH, anti- HDAC5, and anti-ANP antibodies were from Santa Cruz Biotechnology (Santa Cruz, USA). Monoclonal anti – Phospho-p44/42 MAPK and polyclonal anti-MEK1/2 were purchased from Cell Signaling Technology (Boston, USA), monoclonal anti-CAMK2δ from Abnova (Taipei, Taiwan). Hydromount was from National Diagnostics (St. Louis, USA). PD- 98059 (MEK 1/2 innhibitor) was from Calbiochem (Germany). Enhanced chemiluminescence (ECL-plus Western Blotting Detection System) and peroxidase-conjugated antibodies were purchased from Amersham Biosciences (Buckinghamshire, UK). All other reagents were of the highest quality commercially available.

### Human samples

Use of blood samples from patients with acute coronary disease, acute myocardial infarction and hepatic transplant as well as the term of free and informed consent, n: 03182712.2.0000.5149 and n: 00907612.0.0000.5149, respectively, were approved by the Ethics Committee in Research of UFMG-COEP.

### Animals

In this study, we used adult (250 g) and neonatal (3–5 days old) Wistar rats, acquired from Centro de Bioterismo (CEBIO) of the Federal University of Minas Gerais. GPR91^*−/−*^ mice (C57BL/6 background) were provided by Novartis. Animals were maintained on a standard diet and housed under a 12-hour light–dark cycle. All animal experiments were performed in accordance to the *Guide for the Care and Use of Laboratory Animals* published by the US National Institutes of Health (NIH publication No. 85–23, revised 1996).

### Genotyping of mice

Mice were characterized by PCR using genomic DNA extracted from tail biopsies. DNA was digested with the REDExtract-N-Amp Tissue PCR Kit (Sigma-Aldrich). Routine genotyping by PCR was performed using the following primers: The forward primer sequence was 5′ TTA CGC CAC TGG GAA CTG GA3′ and the reverse primer sequence was 5′ TTG ATG GCC TTC TGG GAA CA 3′[56]. Primers for GPR91 were designed using the software Primer3 based on the sequence deposited in the NCBI Nucleotide Bank NM_001001518.1.

### Adult cardiomyocyte isolation

Adult ventricular myocytes were freshly isolated as previously described [59,60] from adult Wistar rats previously treated with 0.066 mg/kg succinate intravenously. Cells were incubated in DMEM and the experiments were carried out at room temperature (22-24°C).

### Primary culture of neonatal cardiomyocytes

Cardiomyocytes were isolated from hearts of 3–5 days old Wistar rats according to the manufacturer’s instructions (Worthington Biochemical Corporation). The rats were anesthetized with sodium pentobarbital (50 mg/kg body weight intraperitoneally), and the beating hearts were removed surgically. Cells were resuspended in Dulbeccos’s Modified Eagle’s Medium (DMEM) supplemented with 10% fetal bovine serum (FBS), 100 units/ml penicillin, 100 μg/ml streptomycin and 0.25 μg/ml anfotericin-b. Cardiomyocytes were plated into fibronectin-coated culture dishes or flasks, and incubated at 37°C in a 5% CO_2_ incubator. Two days after plating, cells were rinsed with DMEM and fed for another 24 h with regular culture medium, now including 20 μg/ml cytosine β-D-arabinofuranoside (ARA-C). The ARA-C was added to the culture medium for 48 h to inhibit growth of non-cardiomyocyte cells. The medium was then replaced with FBS-free DMEM with or without succinate (1 mmol/L) for 12 h and subsequently changed to 10% FBS-containing medium with or without succinate (1 mmol/L) for 24 h. Cardiomyocytes were used in experiments at the fourth day of culture. At this point, there were approximately 5-6 × 10^4^ cardiomyocytes per culture dish, which comprised approximately 95% the total cell population [22,59,61]. The cells were then used for immunofluorescence, western blot or qRT-PCR analysis.

### Preparation of siRNA

Potential target sites within the GPR91 gene were selected and then searched with NCBI Blast to confirm specificity for the receptor. The siRNAs for GPR91, and a siRNA containing the same nucleotides for GPR91 but in a scrambled sequence were prepared by a transcriptional-based method using the Silencer kit, according to the manufacturer’s instructions. The sense and antisense oligonucleotides of siRNA were, respectively: 5′ AAT CTC TAA TGC CAG CCA ATT CCT GTC TC 3′ and 5′ AAA ATT GGC TGG CAT TAG AGA CCT GTC TC 3′. For siRNA studies, day 4 neonatal cardiomyocyte cultures were treated with 100 nM of each siRNA [22]. We used single wall carbon nanotubes (CNT) to deliver siRNA and silence GPR91, as previously described [62]. Cardiomyocytes were incubated at 37°C in an atmosphere of 5% CO_2_ for 48 hours prior to use.

### Western blotting

Cardiomyocytes were harvested as described and protein content was quantified according to Bradford protein assay. For ANP, 50 μg of whole cell proteins were separated by 12% SDS-PAGE. For GAPDH detection, mouse monoclonal anti-GAPDH antibody was used at a dilution of 1:1500. For ERK 1/2 detection, a rabbit polyclonal antibody was used at dilution of 1:500. For ANP detection rabbit polyclonal anti-ANP was used at a dilution of 1:200. For Phospho-p44/42 MAPK (ERK1/2) detection, a rabbit monoclonal antibody was used at a dilution of 1:1000. For CaMKIIδ detection, a mouse monoclonal anti- antibody was used at a dilution of 1:500. The antibody incubation proceeded for 2 h at room temperature. After washing, blots were incubated in HRP-conjugated goat-anti-mouse or rabbit IgG1 secondary antibody at a dilution of 1:5000 at room temperature for 1 h. Immunodetection was carried out using enhanced chemiluminescence [22,59].

### Immunofluorescence

Confocal immunofluorescence was performed as described [22,59]. Briefly, cardiomyocytes were seeded onto 6 well culture dishes, treated with succinate 1 mM and 36 hours later were fixed with 4% paraformaldehyde, permeabilized with PBS 1X/Triton 0.5% and non-specific binding was blocked (PBS, BSA 10%, Triton 0.5%, goat serum 5%) for 1 hour. Cells were then incubated with anti-ANP (1:10), anti α-actinin (1:150), anti-HDAC5 (1:50) or with anti-NFAT (1:50) for 2 hours at room temperature. This was followed by incubation with specific secondary antibodies conjugated with Alexa-Fluo 488 or 633 (1:500) for 1 hour. Images were obtained using Zeiss LSM 510 confocal microscope (Thornwood, USA) [22,59].

### Measurement of intracellular Ca^2+^

Intracellular Ca^2+^ was monitored in individual cardiomyocytes by line scanning and time lapse confocal microscopy as described previously [22,59,60]. Briefly, adult cardiomyocytes were incubated with Fluo-4/AM (6 μmol/L) for 30 minutes at 37°C. Coverslips were transferred to a perfusion chamber on the stage of a Zeiss LSM510 confocal microscope. Cells were electrically stimulated at 1 Hz to produce steady-state conditions. Fluo-4 was excited at 488 nm and observed at 505–550 nm. Increases in Ca^2+^ were expressed as percent increase in Fluo-4 fluorescence over baseline [22,63,64].

### PD 98059 (MEK 1/2 inhibitor), KN93 (Ca^2+^/calmodulin-dependent kinase II inhibitor) and losartan (AT1 angiotensin II receptor antagonist)

10 μmol/L of PD or 1 μmol/L KN93 [39] was added combined of 1 mmol/L succinate to the cells. Losartan was administrated in the drinking water, once a day, for 5 days at doses of 200 mg/l [65].

### Intravenous administration of succinate and measurement of arterial blood pressure

Animals were housed in a temperature-controlled room under a 12 hour light–dark cycle with water and standard rodent chow available *ad libitum*. Rats and mice under ketamine and xylazine anesthesia had the femoral vein catheterized for intravenous injection of succinate (0.066 mg/kg in rats and 0.039 mg/kg in mouse) or PBS. Blood pressure was measured in unanesthetized animals by the tail-cuff method, MAP was recorded in rats for 5 days; at least 15 measurements were made daily with a BP-2000 blood pressure analysis tail-cuff System (isitech Systems). The mean systolic blood pressure and pulse rate were taken for each animal [21,66].

### Real-time PCR

Total RNA was isolated from adult and neonatal cardiomyocytes using TRIzol and cDNA synthesized using SuperScript II kit (Invitrogen). DNA templates were amplified by real time PCR on the StepOnePlus™ Real-Time PCR Systems (Applied Biosystems, CA) using the SYBR green method, as described. Sequence of the primers used are: ANP FW: 5′-GGATTTCAAGAACCTGCTAGA-3′ and RE 5′-CTTCATCGGTC TGCTCGCTCA-3′; BNP5′AGACCACCGCTC TTGTGTGTG-3′ and RE 5′ CTGACC CATACCTACCATGACACC-3′; MHC and GPR91 FW: 5′-TTACGCCACTGGGAACT GGA-3′ and RE 5′-TTGATGGCCTTCTGGGAACA-3 [22,61,67]: FW: 5′-CAG

GCGGTGCTGTCTCTCTAT-3′ and RE: 5′-GGCAGGGCATAACCCTCATA-3′ for α-SkA [61]. Experiments were performed in triplicate for each data point.

### Tissue preparation

After administration for 5 days succinate the rats were weighed, anesthetized (i.p. sodium pentobarbital 80 mg/BW plus ketamine chloridrate 10 mg/BW) and perfused intracardially with Ringer’s solution followed by 10% neutral buffer formalin (NBF). After fixation, the heart was removed, weighted and the relative organ weight was calculated per 100 g of body weight. Fragments of the heart were embedded in paraffin, sectioned (5 μm) and mounted in silanized glass slides. For histological and morphometric studies, the sections were stained with hematoxylin and eosin or Masson’s trichrome [68].

### Morphometry

The quantitative analysis of myocyte cross-sectional height and nuclear diameter were measured in histological sections by using computer-assisted image analysis and the Scion Image software (http://www.scioncorp.com), as previously described [68]. For this purpose, digital images were obtained with a Nikon Coolpix 995 digital camera (Nikon Instruments Inc., Melville, USA).

### HPLC analysis

Serum samples from humans were collected to determine succinate using HPLC as previously described [69]. Mice samples were collected under CO_2_ euthanasia, while human samples were collected from conscious individuals. All subjects had documented coronary atherosclerosis. Accurate details about the inclusion and exclusion criteria for this trial were previously published. All human subjects gave written informed consent, and the study was approved by the Institutional Review Committees at all sites.

### Echocardiography

Animals were anaesthetized using a nose cone with isoflurane at 5% for one minute and the maintenance dose was 1.25%. The anterior chest was shaved, and the animals were placed in supine position on an imaging stage equipped with built-in electrocardiographic electrodes for continuous heart rate monitoring and a proper heating pad to avoid hypothermia. *In vivo* cardiac function was assessed noninvasively using a high-frequency, high-resolution echocardiographic system consisting of a VEVO 2100 ultrasound

Machine equipped with a 30–40 MHz bifrequencial transducer (Visual Sonics, Toronto, Canada). High-resolution images were obtained as previously described [70-73].

### Statistical analysis

Results are expressed as mean values ± S.E., except where otherwise noted. Prism (GraphPad Software, San Diego, CA) and Image J (NIH; Bethesda, MD) were used for data and image analysis, respectively. Statistical significance was tested using One-way ANOVA followed by Bonferroni test, and p value < 0.05 was taken to indicate statistical significance.

## Additional files

Additional file 1: Figure S1. Losartan partially reverted expression of ANP and MYH7 induced by succinate. A. ANP mRNA levels in freshly isolated adult cardiomyocytes from control, control with losartan, succinate treated and succinate treated in the presence of losartan rats. B. MYH7 mRNA levels in freshly isolated adult cardiomyocytes from control, control with losartan, succinate treated and succinate treated in the presence of losartan rats. (control n = 6, control + losartan n = 3, succinate n = 8 and succinate + losartan n = 5, ***p < 0.001). Additional file 2: Figure S2. Succinate induces hypertrophy concentration dependent. A. Graph show different concentrations of succinate, and cellular width (500 ± 12 μm^2^ for control cells; 682 ± 20 μm^2^ for cells treated with 25 mM succinate; 800 ± 15 μm^2^ for 0.5 mM succinate, 1000 ± 15 μm^2^ for 0.75 mM succinate, 1041 ± 25 μm^2^ for 1 mM succinate, 1083 ± 13 μm^2^ for 1.5 mM succinate, 1080 ± 10 μm^2^ for 2 mM succinate, 1085 ± 20 μm^2^ for 2.5 mM succinate). Additional file 3: Figure S3. Succinate does not activate NFAT. Top: representative images of cardiomyocytes immunostained with antibodies against NFAT (green), α-actinin (red) and DAPI (blue). Scale bar represents 10 μm. Bottom: Quantification of NFAT nuclear fluorescence (p > 0.05, n = 45 cells). Additional file 4: Figure S4. Silencing of GPR91 prevented the translocation of HDAC5 and the increase in cellular area induced by succinate. A. Representative images of cardiomyocytes immunostained with antibodies against HDAC5 (red) and α-actinin (green). Silencing of GPR91 prevented the translocation of HDAC5 from the nucleus to the cytosol . Scale bar represents 10 μm. B. Quantification of the nuclear fluorescence for HDAC5 (***p < 0.001), n = 30 cells). C. Quantification of the cellular area. (**p < 0.01). Additional file 5: Figure S5. Intravenous administration of succinate alters phosphorylation levels of ERK1/2. Top: representative immunoblot of whole-cell protein lysates from ventricular cardiomyocytes probed with anti-phospho ERK1/2 at Thr ^202/^Tyr^204^ site and anti-ERK/1/2. Bottom: Bar graph shows that succinate significantly increases ERK1/2 phosphorylation levels. These results represent the mean ± S.E. of three separate experiments (**p < 0.01). Additional file 6: Figure S6. Intravenous administration of succinate alters global Ca^2+^ transients in adult ventricular cardiomyocytes. A - D. Global Ca^2+^ transients in freshly isolated adult rat cardiomyocytes. Ca_i_^2+^ was monitored with Fluo-4/AM using confocal linescanning microscopy. Cells were examined after intravenous administration of succinate and compared to control. Images are pseudocolored according to the color scale shown at the right of panel A. Tracing under each panel shows the percent increase in fluorescence relative to baseline, and is representative of the indicated cell. E- F. Summary of succinate effects on Ca^2+^ transient amplitude. Ca^2+^ kinetics of decay (presented as T90) was significantly faster in cells after succinate treatment when compared to controls. (***p < 0.001, *p < 0.05). Additional file 7: Figure S7. Succinate increases in the serum of mice subjected to ischemia and reperfusion. **A**. The bar graph shows the mean concentration of succinate in the serum of control mice, sham and animals subjected to liver ischemia and reperfusion, (n = 3, p < 0.001). Additional file 8: Table S1. Additional health conditions and characteristics of the cardiac patients.

## Abbreviations

ANP: Atrial natriuretic peptide
BNP: Brain natriuretic peptide
CaMKIIδ: Calcium/calmodulin dependent protein kinase IIδ
ERK 1/2: Extracellular signal-regulated kinase 1/2
GSK3: Glycogen synthase kinase 3
HDAC5: Histone deacetylase 5
JNK: c-Jun N-terminal kinase
MAP: Mean arterial pressure
MYH7: β myosin heavy chain
NFAT: Nuclear factor of activated T-cells
NT-pro-BNP: N-terminal pro-B-type natriuretic peptide
phospho-ERK 1/2: Phosphorylated extracellular signal-regulated kinase 1/2
WT: Wild type
