# Supplementary figures and images for: Succinate causes pathological cardiomyocyte hypertrophy through GPR91 activation
Source: Cell Commun Signal. 2014 Dec 24;12:78. doi: 10.1186/s12964-014-0078-2 (PMC4296677; doi:10.1186/s12964-014-0078-2)

**A**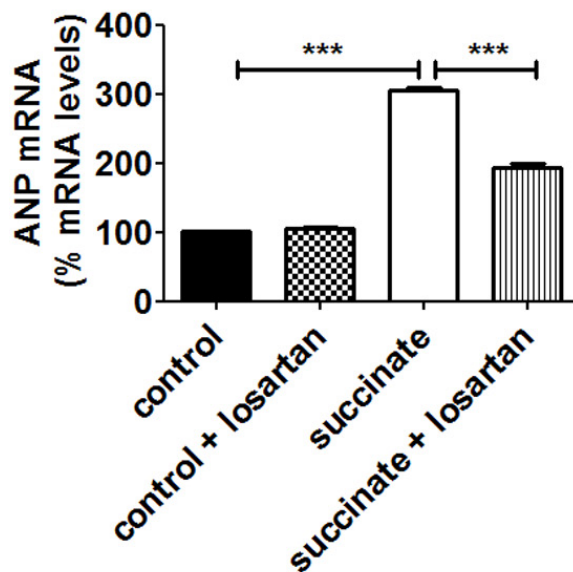**B**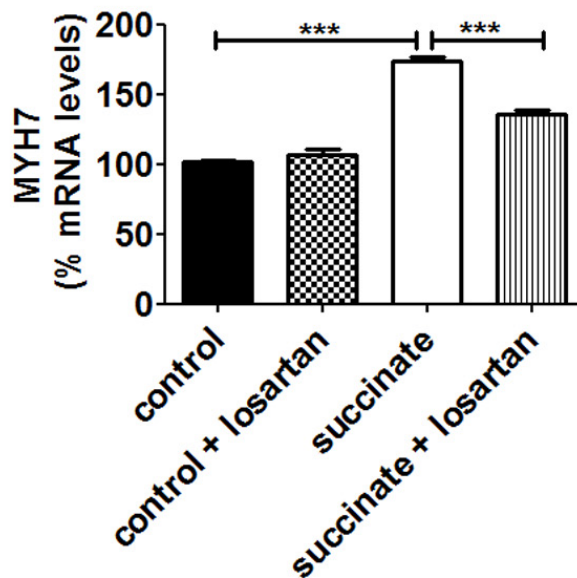

**Aguiar, C. J. *et. al.*  
supplementary 1**

Supplement: Additional file 1: Figure S1. — Losartan partially reverted expression of ANP and MYH7 induced by succinate. A. ANP mRNA levels in freshly isolated adult cardiomyocytes from control, control with losartan, succinate treated and succinate treated in the presence of losartan rats. B. MYH7 mRNA levels in freshly isolated adult cardiomyocytes from control, control with losartan, succinate treated and succinate treated in the presence of losartan rats. (control n = 6, control + losartan n = 3, succinate n = 8 and succinate + losartan n = 5, ***p < 0.001). [file 12964_2014_78_MOESM1_ESM.pdf]

**A**

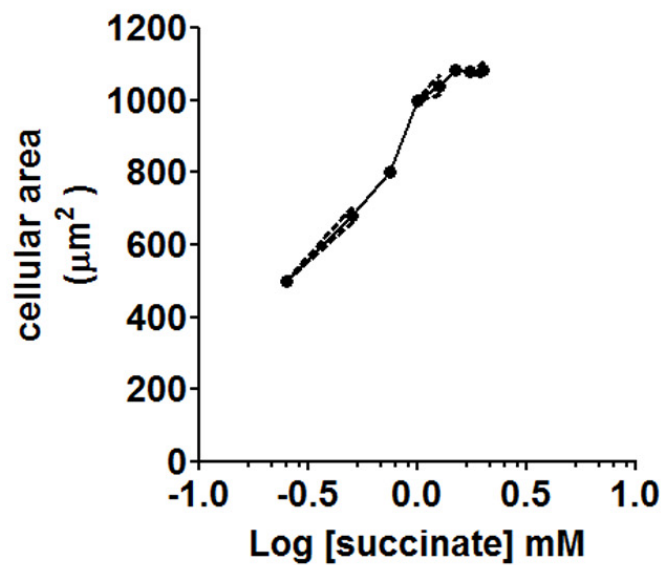

**Aguiar, C. J. *et. al.*  
supplementary 2A**

Supplement: Additional file 2: Figure S2. — Succinate induces hypertrophy concentration dependent. A. Graph show different concentrations of succinate, and cellular width (500 ± 12 μm2 for control cells; 682 ± 20 μm2 for cells treated with 25 mM succinate; 800 ± 15 μm2 for 0.5 mM succinate, 1000 ± 15 μm2 for 0.75 mM succinate, 1041 ± 25 μm2 for 1 mM succinate, 1083 ± 13 μm2 for 1.5 mM succinate, 1080 ± 10 μm2 for 2 mM succinate, 1085 ± 20 μm2 for 2.5 mM succinate). [file 12964_2014_78_MOESM2_ESM.pdf]

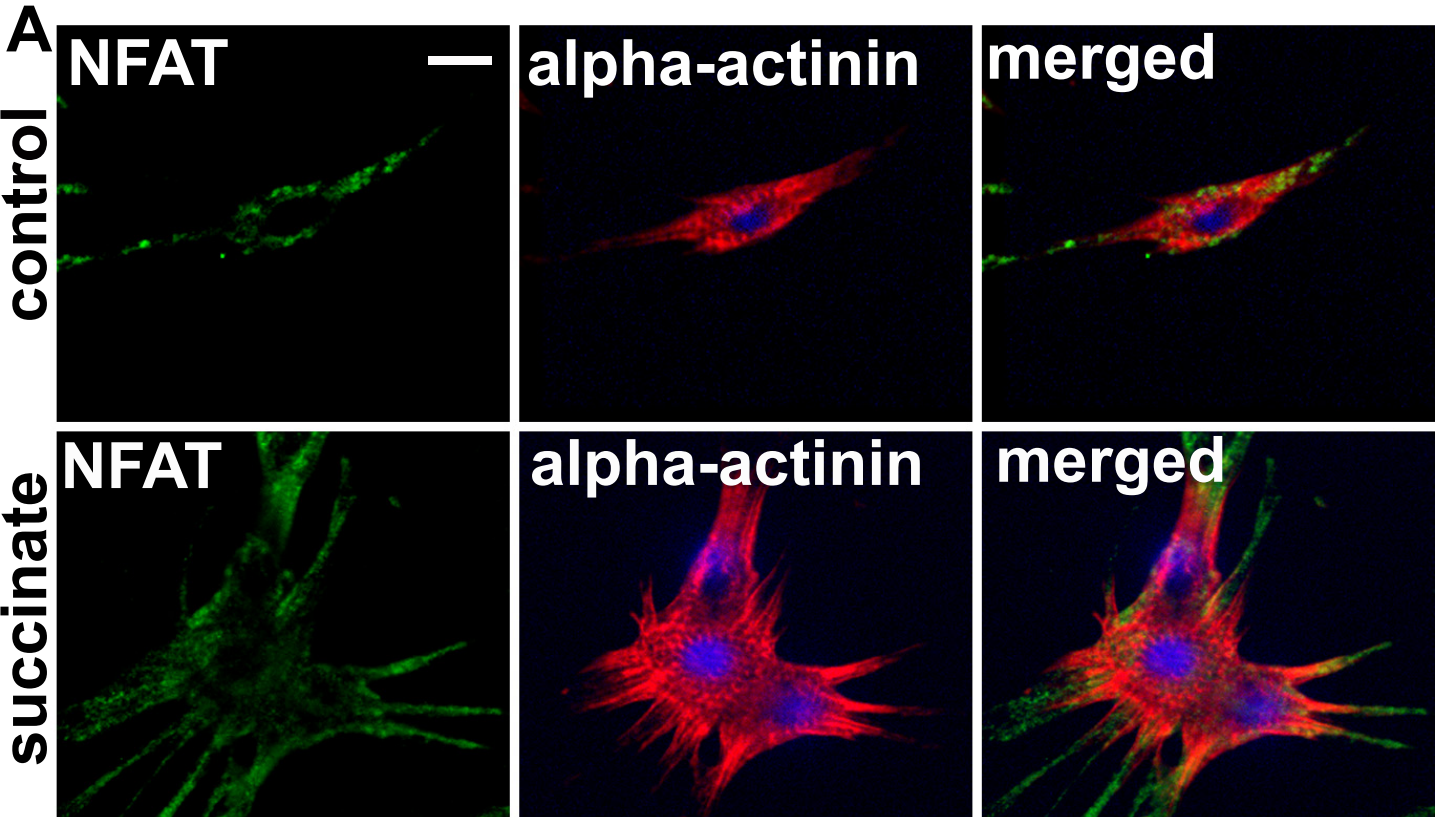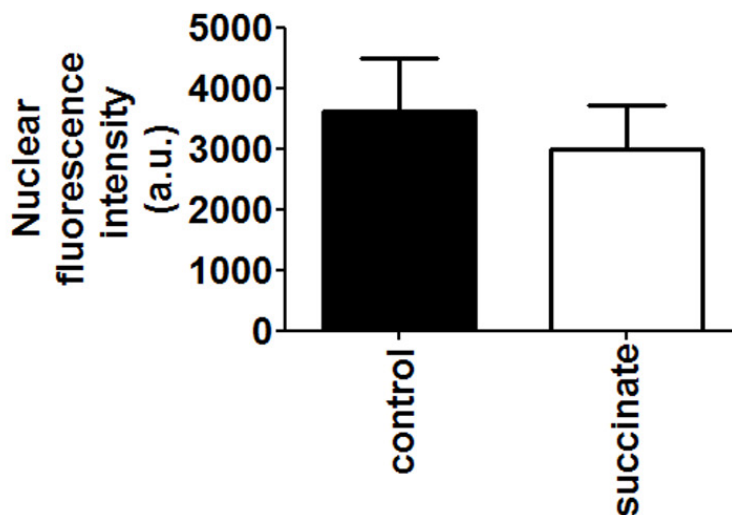

**Aguiar, C. J. *et. al.*  
supplementary 3**

Supplement: Additional file 3: Figure S3. — Succinate does not activate NFAT. Top: representative images of cardiomyocytes immunostained with antibodies against NFAT (green), α-actinin (red) and DAPI (blue). Scale bar represents 10 μm. Bottom: Quantification of NFAT nuclear fluorescence (p > 0.05, n = 45 cells). [file 12964_2014_78_MOESM3_ESM.pdf]

**A**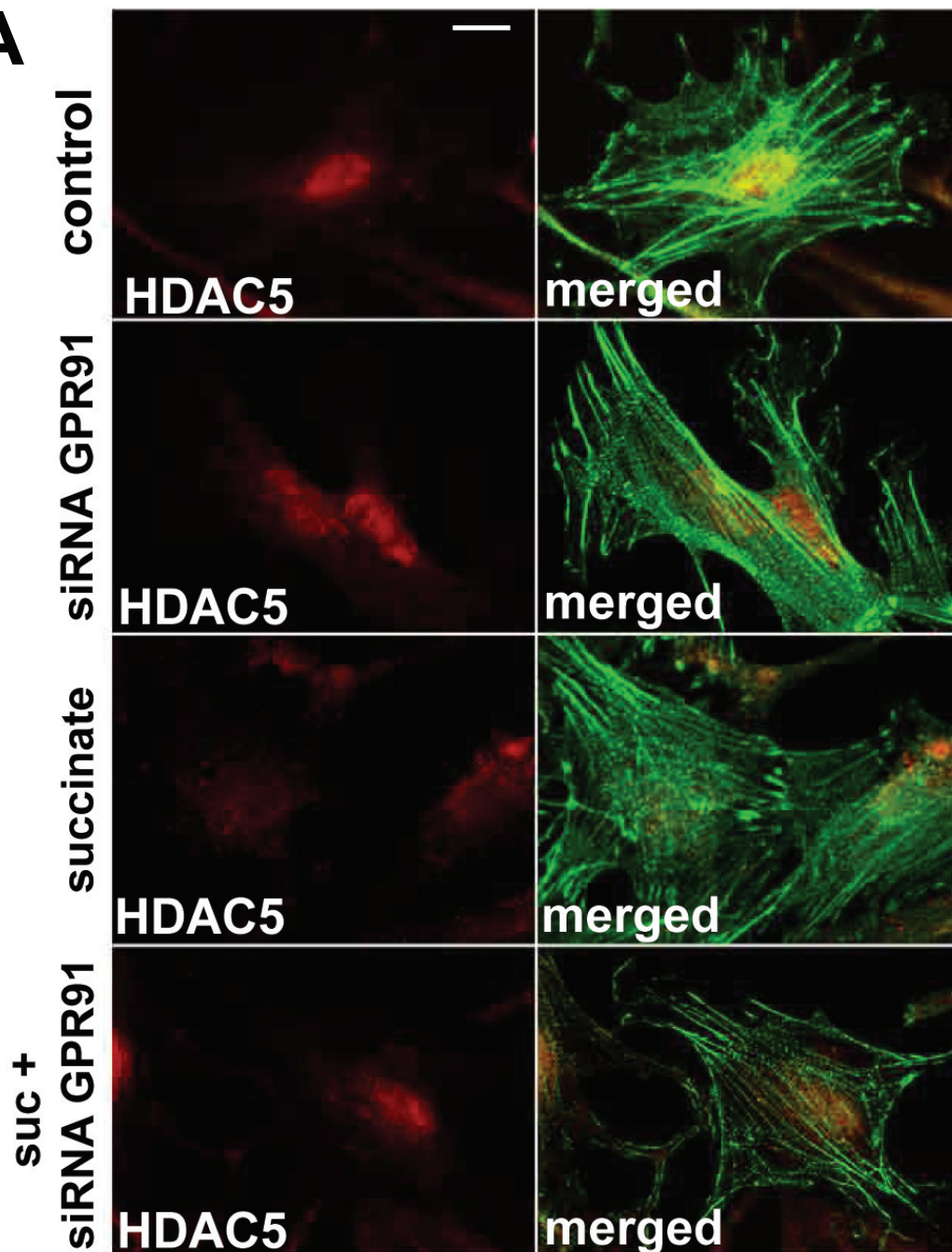

**Aguiar, C . J. *et. al.*  
supplementary 4A**

**B**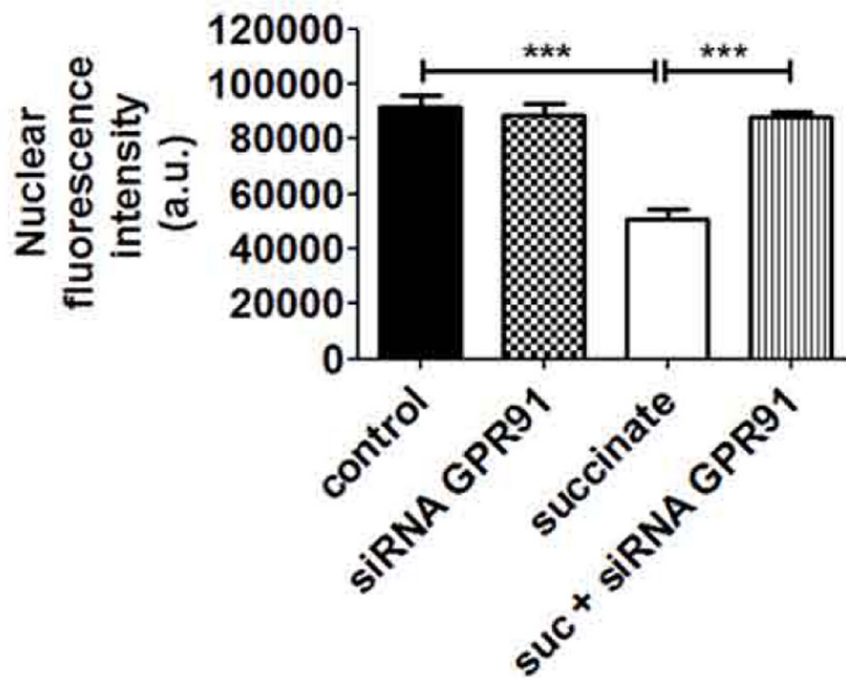**C**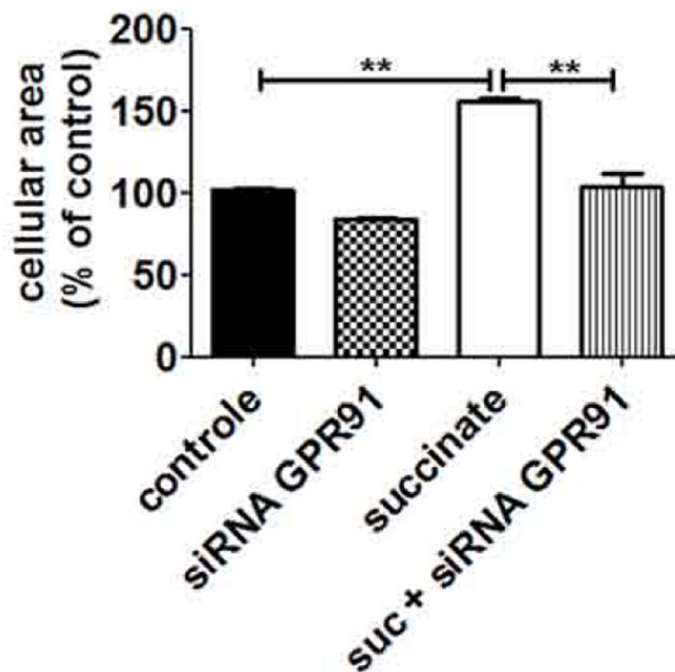

**Aguiar, C. J. et. al.**  
**supplemental 4B & C**

Supplement: Additional file 4: Figure S4. — Silencing of GPR91 prevented the translocation of HDAC5 and the increase in cellular area induced by succinate. A. Representative images of cardiomyocytes immunostained with antibodies against HDAC5 (red) and α-actinin (green). Silencing of GPR91 prevented the translocation of HDAC5 from the nucleus to the cytosol . Scale bar represents 10 μm. B. Quantification of the nuclear fluorescence for HDAC5 (***p < 0.001), n = 30 cells). C. Quantification of the cellular area. (**p < 0.01). [file 12964_2014_78_MOESM4_ESM.pdf]

**A**

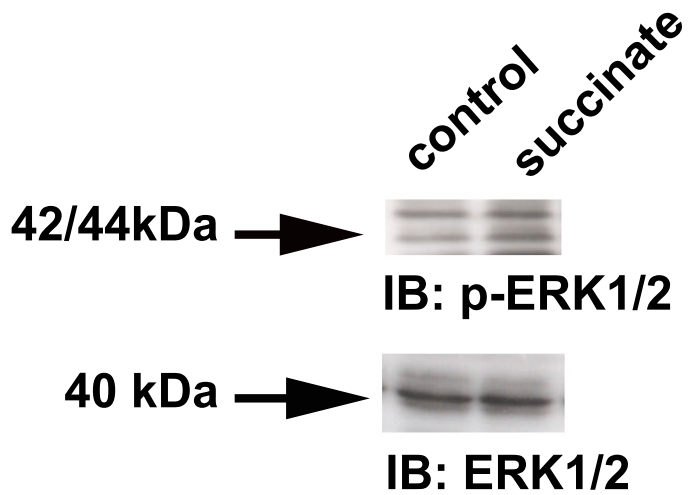

**B**

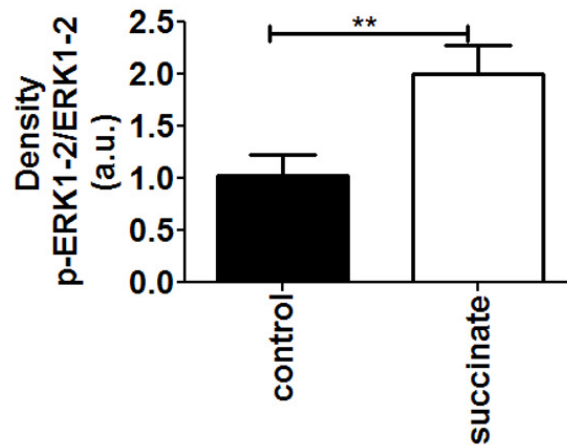

**Aguiar, C. J. *et. al.***  
**supplementary 5A & B**

Supplement: Additional file 5: Figure S5. — Intravenous administration of succinate alters phosphorylation levels of ERK1/2. Top: representative immunoblot of whole-cell protein lysates from ventricular cardiomyocytes probed with anti-phospho ERK1/2 at Thr 202/Tyr204 site and anti-ERK/1/2. Bottom: Bar graph shows that succinate significantly increases ERK1/2 phosphorylation levels. These results represent the mean ± S.E. of three separate experiments (**p < 0.01). [file 12964_2014_78_MOESM5_ESM.pdf]

**A**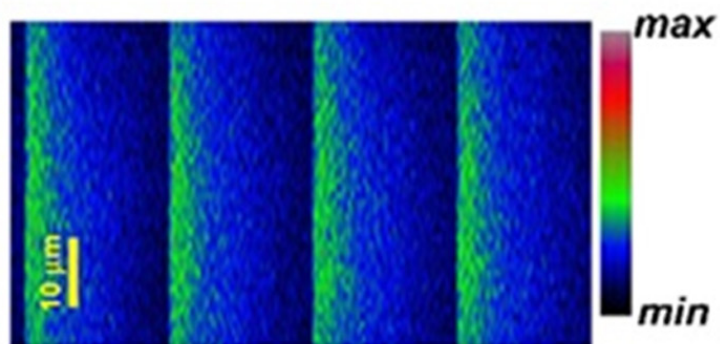**B**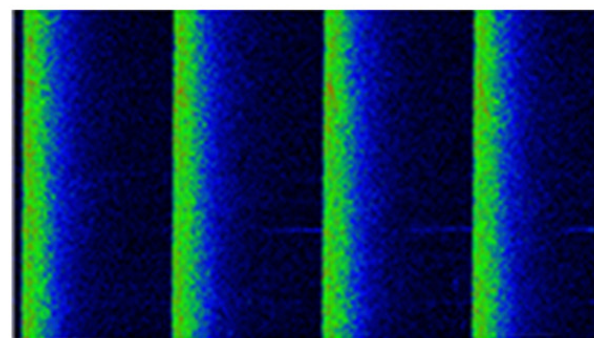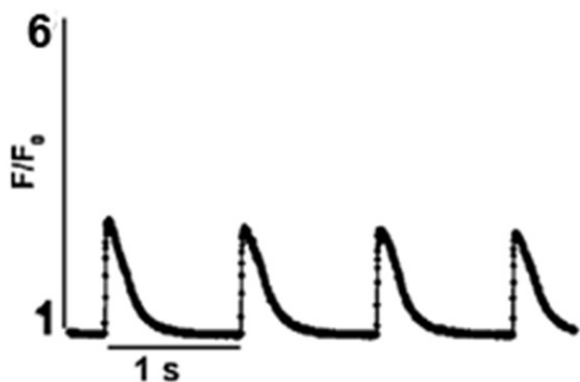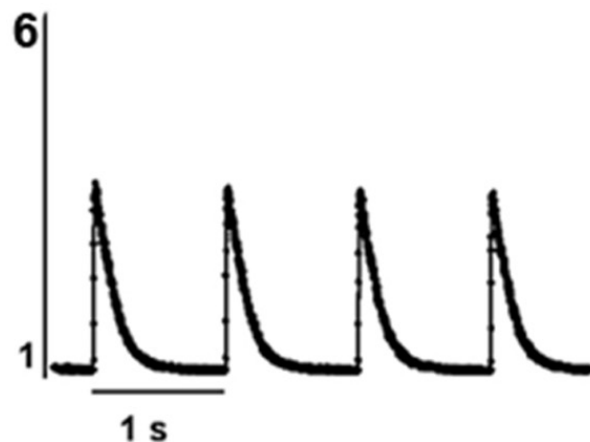**C**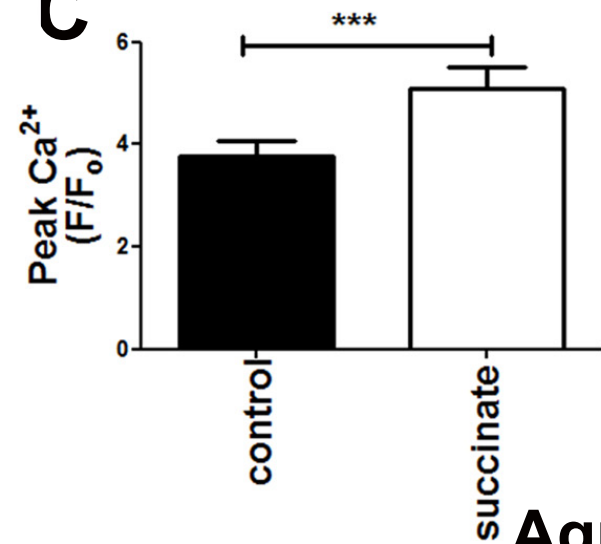**D**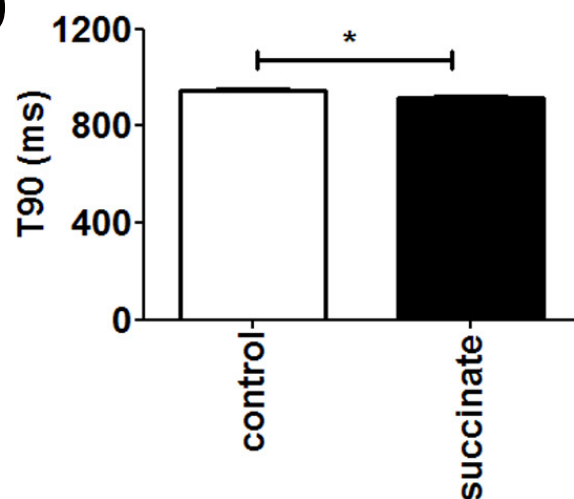

**Aguiar, C. J. *et.al.***  
**supplementary 6A, B, C & D**

Supplement: Additional file 6: Figure S6. — Intravenous administration of succinate alters global Ca2+ transients in adult ventricular cardiomyocytes. A - D. Global Ca2+ transients in freshly isolated adult rat cardiomyocytes. Cai 2+ was monitored with Fluo-4/AM using confocal linescanning microscopy. Cells were examined after intravenous administration of succinate and compared to control. Images are pseudocolored according to the color scale shown at the right of panel A. Tracing under each panel shows the percent increase in fluorescence relative to baseline, and is representative of the indicated cell. E- F. Summary of succinate effects on Ca2+ transient amplitude. Ca2+ kinetics of decay (presented as T90) was significantly faster in cells after succinate treatment when compared to controls. (***p < 0.001, *p < 0.05). [file 12964_2014_78_MOESM6_ESM.pdf]

**A**

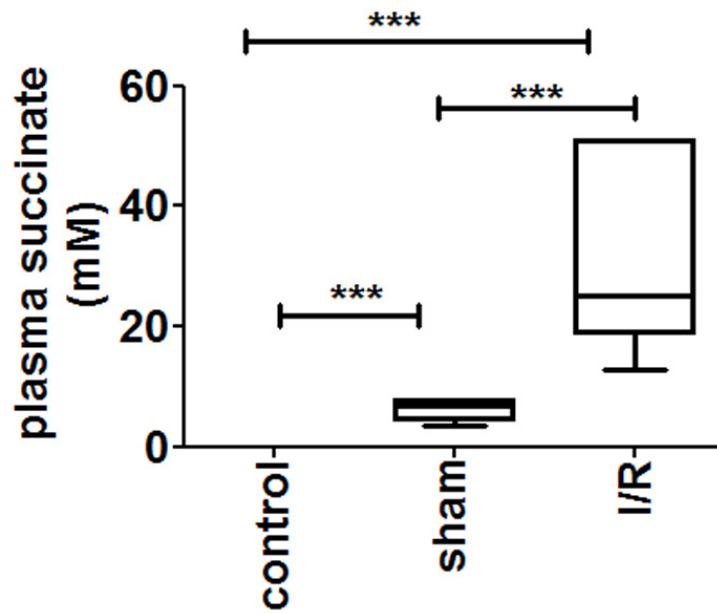

**Aguiar, C. J. *et. al.*  
supplementary 7**

Supplement: Additional file 7: Figure S7. — Succinate increases in the serum of mice subjected to ischemia and reperfusion. A. The bar graph shows the mean concentration of succinate in the serum of control mice, sham and animals subjected to liver ischemia and reperfusion, (n = 3, p < 0.001). [file 12964_2014_78_MOESM7_ESM.pdf]
