# Supplementary material for: Succinate causes pathological cardiomyocyte hypertrophy through GPR91 activation
Source: Cell Commun Signal. 2014 Dec 24;12:78. doi: 10.1186/s12964-014-0078-2 (PMC4296677; doi:10.1186/s12964-014-0078-2)
Supplement: Additional file 8: Table S1. — Additional health conditions and characteristics of the cardiac patients. [file 12964_2014_78_MOESM8_ESM.pdf]

Additional file 8: Table S1. Additional health conditions and characteristics of the cardiac patients

Patient 1
Hypertension
Aortic aneurysm

Patient 2
Hypertension
Acute myocardial infarction without ST-segment elevation

Patient 3
Congestive heart failure
Alcoholic and smoker

Patient 4
Diabetes mellitus
Congestive heart failure
LVEF 24%
Peripheral vascular insufficiency
Left ventricular thrombus

Patient 5
Hypertension
Diabetes mellitus
Retinopathy
Nephropathy
Congestive heart failure
LVEF 34%

Patient 6
Hypertension
Smoker
Unstable angina

Patient 7
Congestive heart failure
LVEF 24%
Smoker
Left ventricular thrombus

Patient 8
Diabetes mellitus
Hypertension
Smoker
Smoker

Patient 9
Hypertension
Peripheral vascular insufficiency
Congestive heart failure
LVEF 30%
